# Investigating the Association Between Volatile Organic Compounds and Preterm Birth in Detroit, Michigan: Protocol for the Center for Leadership in Environmental Awareness and Research Birth Cohort Study

**DOI:** 10.2196/87272

**Published:** 2026-06-24

**Authors:** Andrea E Cassidy-Bushrow, Emily Leydet, Albert M Levin, Mei Lu, Jennifer K Straughen

**Affiliations:** 1 Department of Public Health Sciences, Henry Ford Hospital, 1 Ford Place, Detroit, MI, 48202, United States, 1 3138746097; 2 Department of Pediatrics and Human Development, College of Human Medicine, Michigan State University, East Lansing, MI, United States; 3 Department of Epidemiology and Biostatistics, College of Human Medicine, Michigan State University, East Lansing, MI, United States; 4 Department of Women's Health Services, Henry Ford Hospital, Detroit, MI, United States; 5 Department of Obstetrics, Gynecology and Reproductive Biology, College of Human Medicine, Michigan State University, East Lansing, MI, United States

**Keywords:** birth cohort, volatile organic compounds, preterm birth, placenta, inflammation

## Abstract

**Background:**

Preterm birth (PTB), or birth before 37 weeks of gestation, remains a significant public health issue in the United States, particularly in Detroit, Michigan. Growing evidence suggests that volatile organic compounds (VOCs), aromatic or chlorinated organic compounds that vaporize readily, may influence PTB risk. However, much of this prior work is limited by indirect VOC exposure estimates (eg, assignment based on maternal residential address), single-point or cumulative exposure estimates during pregnancy, or limited consideration of potential mechanistic factors.

**Objective:**

The Center for Leadership in Environmental Awareness and Research (CLEAR) birth cohort has been designed to test the hypotheses that prenatal VOC exposures, measured as VOC metabolites in maternal urine, increase the risk of PTB; that VOC exposures are associated with maternal inflammation and placental function measures; that associations between prenatal VOC exposures and PTB may be mediated by these maternal inflammation and placental function measures; and that there are neighborhood-level factors that may increase the risk of VOC exposure during pregnancy.

**Methods:**

A prospective cohort of 1075 pregnant patients receiving prenatal care at Henry Ford Health will be recruited. Pregnant patients residing in Detroit or receiving prenatal care at a Detroit-based Henry Ford Health women’s health clinic are eligible. Pregnant patients are followed until delivery. Up to 3 urine and blood samples (collected during early, mid, and late pregnancy) are obtained for measurement of VOC metabolites and inflammatory biomarkers, respectively. The placenta is obtained after delivery for epigenomic and transcriptomic measurement. Surveys are administered to pregnant participants to assess a variety of lifestyle, psychosocial, medical, residential, and other factors. Address information collected from both surveys and electronic medical records across pregnancy will be used to identify potential sources of VOC exposure. The electronic medical record is used to obtain medical and delivery data, including infant sex, date of delivery, and gestational age (GA) at delivery. PTB, the primary study outcome, is defined as GA at delivery <37 weeks. A nested case-control approach (frequency matching PTB cases 1:1 with full-term controls [GA at delivery ≥37 weeks] based on infant sex and maternal race) will be applied. Statistical methods, including logistic regression, linear mixed methods, and geographically weighted regression models, as well as chemical mixture approaches, will be used.

**Results:**

Funding began September 2022 and recruitment commenced November 2023. Through April 22, 2026, a total of 468 pregnant patients have consented to participate in the CLEAR birth cohort, and recruitment is ongoing.

**Conclusions:**

The CLEAR cohort will provide novel data on the role of VOCs during pregnancy in the risk of PTB. Additionally, the role of VOC exposures during pregnancy in maternal inflammation and placental function will be examined. Finally, potential sources of VOC exposures, which could be targets for environmental remediation, will be identified.

## Introduction

Preterm birth (PTB; gestational age [GA] at delivery <37 weeks) is associated with both immediate and long-term health consequences for the infant, including increased risks of infant mortality, neurodevelopmental delays, obesity, and other chronic diseases. The United States consistently reports higher PTB rates than other high-income nations, with certain cities (particularly those in the postindustrial “rust belt” along the Great Lakes waterways) exceeding the national average. Among these, Detroit, Michigan, has the highest PTB rate of all large US cities, significantly surpassing the national rate (15.6% vs 10.4%) [1,2]. Despite this, few studies have sufficiently examined the contribution of environmental contaminants such as volatile organic compounds (VOCs)—prevalent in urban areas such as Detroit—to PTB risk. Thus, Detroit, which may be “...paradigmatic of communities around the world that depend on heavy industry” [3], may be an ideal setting for studying such associations [4].

VOCs are a vast group of aromatic or chlorinated organic compounds that vaporize readily at room temperature. Common VOCs include benzene, toluene, ethylbenzene, and xylene (collectively known as BTEX), trichloroethylene, and tetrachloroethylene (also known as perchloroethylene). There is growing evidence demonstrating that BTEX, trichloroethylene, and perchloroethylene increase the risk of PTB [4-13]. In our previous study of Detroit pregnancies, every 5-unit increase in airshed BTEX level was associated with 1.54 times higher odds of PTB (95% CI 1.25-1.89) [14]. Most of these prior studies are limited by reliance on estimated exposures and thus are subject to bias due to potential exposure misclassification.

Beyond enhancing our understanding of the association between VOCs and PTB risk, additional investigations into the mechanisms by which VOCs may lead to PTB are needed, as such studies may inform future prevention efforts. On the basis of a small but growing body of evidence, we hypothesize that VOCs may impact maternal systemic inflammation and DNA methylation. Both benzene and perchloroethylene can alter cytokine levels [15,16]. Similarly, VOC exposure alters DNA methylation patterns and gene expression in nontarget tissue (ie, blood) [17-22]. Our recently published data from Detroit pregnancies corroborate these findings, with higher airshed BTEX levels being associated with higher midpregnancy interleukin-1 beta and tumor necrosis factor-alpha levels [23] and differentially methylated regions [24] in maternal blood. In the Lifestyle-Immune System-Allergy birth cohort, maternal perchloroethylene exposure was associated with fewer interferon-gamma–producing T-cells [25]. Finally, in the EDEN (*Etude des Déterminants pré et post natals précoces du développement psychomoteur et de la santé de l'ENfant*) birth cohort study, higher maternal benzene exposure was associated with decreased cord blood Cluster of Differentiation 4+ Cluster of Differentiation 25+ T-regulatory cells [26]. Despite these suggestive findings, there is a need for additional studies examining direct VOC exposure (rather than estimates based on residential address or occupation), at multiple time points across pregnancy, and which incorporate a biologically-relevant tissue—the placenta.

**Figure 1.**
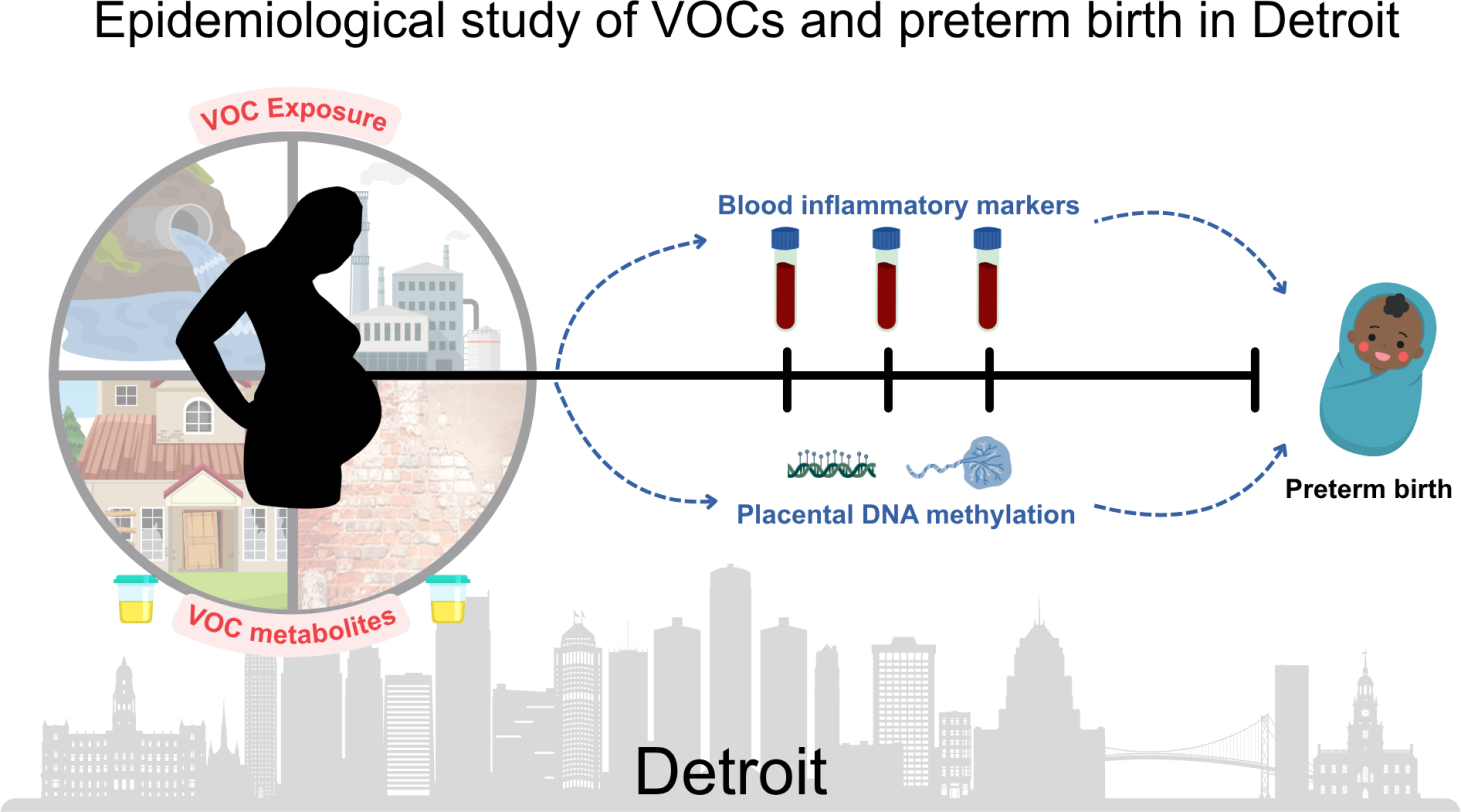
Overview of the study aims. VOC: volatile organic compound.

To address these gaps, the Center for Leadership in Environmental Awareness and Research (CLEAR) birth cohort study, which is part of a National Institute of Environmental Health Sciences–funded Superfund Research Program P42 Center and focused on 6 specific VOCs (Table 1) and PTB, is underway. The CLEAR P42 Center consists of 2 engineering projects (projects 1 and 2), 3 biomedical projects (projects 3, 4, and 5), and 5 cores (administrative core, chemical analysis core, community engagement core, research experience and training coordination core, and data management and analytics core). The specific aims of the CLEAR birth cohort study are to examine whether VOC metabolite levels in maternal urine are associated with PTB, maternal systemic inflammation, and changes in placental function (as measured by DNA methylation and transcriptional gene expression). Exploratory work considering maternal inflammation and placental function changes as potential mediators of associations between VOCs and PTB is planned. Finally, potential sources of VOC exposures will be examined. An overview of the study aims is shown in Figure 1.

**Table 1.** Volatile organic compound (VOC) metabolites to be measured in urine samples from participants in the Center for Leadership in Environmental Awareness and Research birth cohort study and major sources of exposure.

Parent VOC	Metabolite	Major sources
Trichloroethylene	N-acetyl-S-(1,2-dichlorovinyl)-L-cysteine and N-acetyl-S-(2,2-dichlorovinyl)-L-cysteine	Extraction solvent; dry cleaning; paints or strippers, adhesives, lubricants, varnish, and pesticides; and cold metal cleaners [27]
Tetrachloroethylene	N-acetyl-S-(trichlorovinyl)-L-cysteine	Dry cleaning industry and liquid waste from metal degreasing [28]
Benzene	S-phenylmercapturic acid and trans,trans-muconic acid	Auto exhaust, tobacco smoke, automobile service stations, and industrial emissions [29]
Toluene	Benzylmercapturic acid	Gasoline or fuel production; paints; paint thinners; fingernail polish, lacquers, adhesives, and rubber; and tobacco [30]
Ethylbenzene	Phenylglyoxylic acid	Styrene production, cleaners, paints, fuel, pesticides, solvents, and tobacco smoke [31,32]
Xylene	Dimethylphenyl mercapturic acid, 2MHA^a^, and 3MHA+4MHA	Auto exhaust; industrial solvent; airplane fuel; gasoline; paint, varnish, or shellac; rust prevention products; and cigarettes [33]

a MHA: methylhippuric acid.

## Methods

### Participants and Recruitment

CLEAR is a prospective longitudinal birth cohort study that will follow a target sample of 1075 pregnant patients in the Detroit area from pregnancy through delivery. Recruitment for CLEAR began in November 2023 and is ongoing. Through June 2025, participants were enrolled into both the CLEAR birth cohort protocol and a birth cohort focused on asthma and allergy [34-36] under a combined protocol. To reduce participant burden and focus on pregnant patients in Detroit, CLEAR-only enrollment began in July 2025, which reduced data and sample collection requirements. The CLEAR protocol is identical for those enrolled both before and after June 2025.

To be eligible for the CLEAR study, participants must be aged ≥18 years, English-speaking, either receiving prenatal care at a Detroit-based Henry Ford Health (HFH) Women’s Health Services (WHS) clinic or residing in Detroit and receiving care at any HFH WHS clinic, and planning to deliver at an HFH hospital. Pregnant patients are eligible to join the study through 28 weeks of gestation. Because the baseline risk of PTB is higher in multiple gestation pregnancies [37], only singleton pregnancies are eligible for CLEAR. However, not all pregnant patients know they are expecting multiples at the time of study recruitment; participants who consent to the study but are later determined to have multiple gestations are removed from the study.

Potentially eligible pregnant patients are identified through the HFH electronic medical record (EMR) system, Epic (Epic Systems Corp). Eligible patients are entered into a study-specific web-based database. This database is an internally developed HFH platform used for recruitment tracking, study communications, appointment scheduling, sample tracking, and incentive management.

CLEAR recruitment uses multiple approaches in an effort to be broadly appealing and accessible to all eligible patients. The primary points of introduction to the study are the following: (1) in-person recruitment at a prenatal care appointment, (2) a recruitment message via the HFH online patient portal (MyChart; Epic Systems Corp), and (3) a mailed postal letter. Eligible patients may be contacted up to 8 additional times using a combination of texting, telephone calls, and email over a 6-week period or until they exit the eligibility window. Patients who decline participation or cannot be reached are assigned a final disposition code (eg, declined to participate, with a brief reason if available, or no contact, respectively) in the CLEAR database, after which no further contact is made.

Patients who express interest and are deemed eligible are sent a consent and Health Insurance Portability and Accountability Act (HIPAA) authorization form by email through the CLEAR Research Electronic Data Capture (REDCap) database. Study data are collected and managed using REDCap electronic data capture tools hosted at HFH [38,39]. REDCap is a secure, web-based software platform designed to support data capture for research studies, providing (1) an intuitive interface for validated data capture, (2) audit trails for tracking data manipulation and export procedures, (3) automated export procedures for seamless data downloads to common statistical packages, and (4) procedures for data integration and interoperability with external sources. Each participant is assigned the same study ID in both the CLEAR REDCap and the CLEAR web-based databases. Upon electronic signature of the CLEAR consent and HIPAA form, participants are automatically emailed a copy of the consent and HIPAA authorization form. CLEAR staff is simultaneously notified to review the form for completeness and update the participant’s status in the CLEAR web database to indicate enrollment.

### Ethical Considerations

This study was approved by the HFH Institutional Review Board (13198 and 13306). Eligible pregnant participants provide written informed consent and HIPAA authorization for both their and their baby’s participation. The consent is completed electronically via REDCap, which has previously been shown to be feasible and compliant in clinical research [40]. Participants are provided incentives (in the form of a gift card) for the study activities that they complete. Appropriate data use agreements and material transfer agreements have been obtained to share data and biospecimens across CLEAR projects and cores.

### Data and Sample Collection

#### Surveys

**Figure 2.**
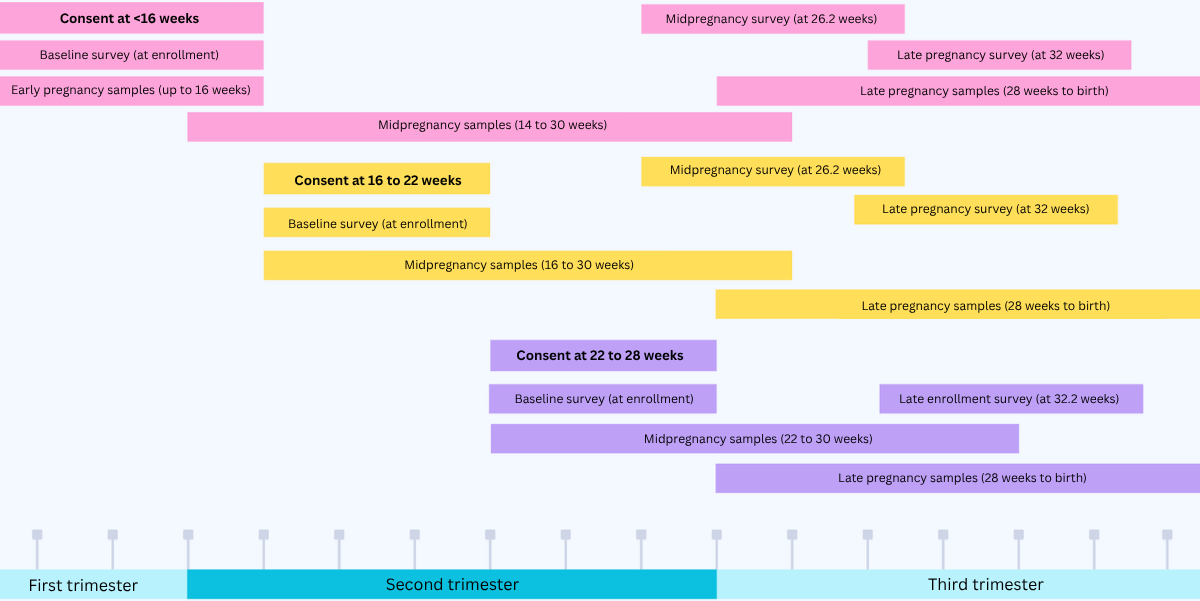
Overview of the study activities based on the gestational age at which a participant provides informed consent. Study activities for participants who provided informed consent at <16 weeks are shown in pink, those who provided informed consent at 16 to 22 weeks are shown in yellow, and those who provided informed consent at >22 to 28 weeks are shown in purple.

After consenting, study questionnaires are sent via email or text, based on the participant’s GA, through REDCap (Figure 2). Surveys are administered up to 3 times during pregnancy: immediately after consent (baseline), in midpregnancy, and in late pregnancy (Figure 2). Participants entering the study “late” (after 22 weeks of gestation) complete the baseline survey and a combined mid- and late-pregnancy survey. Participants are allotted 2 weeks to complete each survey; deadlines have been shown to increase response rates in other settings [41,42]. If a participant delivers before completing the late pregnancy survey, an “early delivery” survey is sent that includes questions from the late pregnancy survey that are unlikely to be influenced by the early delivery (eg, family history of disease; Table 2). The early delivery survey is manually triggered by study staff and sent to the participant based on whether the participant and newborn have been discharged from the hospital or neonatal intensive care unit to avoid overburdening the family during a potentially stressful time.

**Table 2.** Survey and electronic medical record data planned to be collected as part of the Center for Leadership in Environmental Awareness and Research study.

Data elements	Surveys					Electronic medical record
	Baseline	Midpregnancy	Late pregnancy	Late enrollment^a^	Early delivery^b^	
Maternal demographics						
Date of birth						✓
Race and ethnicity	✓					✓
Education	✓					
Employment status	✓					
Occupation	✓					
Marital status	✓					
Place of birth	✓					
Health insurance	✓					✓
Financial resources	✓					
Food insecurity	✓					
Address information (frequently visited locations)			✓	✓		✓
Prenatal health						
Nausea and vomiting	✓		✓			
Blood pressure						✓
Prepregnancy weight	✓					✓
Weight						✓
Height	✓					✓
Medications and supplements used			✓	✓		✓
Dental health			✓	✓		
Infections and illnesses			✓	✓	✓	✓
Maternal medical history						
Chronic conditions						✓
Weight at birth	✓					
Previous pregnancies						
Parity						✓
Gravidity						✓
Previous preterm birth						✓
History of pregnancy complications						✓
Maternal lifestyle						
Physical activity [43]	✓	✓	✓	✓		
Smoking or tobacco use			✓	✓	✓	✓
Alcohol use			✓	✓	✓	✓
Sleep		✓		✓		
Personal care products			✓	✓	✓	
Dietary screener [44,45]			✓	✓		
Pet ownership	✓					
Maternal psychosocial health						
Edinburgh Postnatal Depression Scale [46]	✓^c^	✓	✓	✓		
Perceived Stress Scale [47]	✓	✓	✓	✓		
Confusion, Hubbub, and Order Scale [48]	✓					
PROMIS^d^ Global Physical and Mental Health [49]	✓		✓	✓		
PROMIS Depression Short Form [50]	✓		✓	✓		
Brief Resilience Scale [51]	✓					
Connor-Davidson Resilience Scale [52]	✓					
Broad Autism Phenotype [53]	✓					
PROMIS Informational Support		✓		✓		
PROMIS Emotional Support [54]		✓		✓		
PROMIS Instrumental Support		✓		✓		
Household factors						
Type of housing	✓					
Basement	✓					
Number of inhabitants	✓					
Cleaning habits	✓					
Mold and mildew	✓					
Heating and cooling	✓					
Fresh air	✓					
Air purifiers or filters	✓					
Paternal demographics						
Date of birth	✓					
Race and ethnicity	✓					
Education	✓					
Location of birth	✓					
Paternal lifestyle and health history						
Height	✓					
Weight	✓					
Smoking	✓					
Baby’s family health history			✓	✓	✓	
Delivery information						
Date of delivery						✓
Gestational age at delivery						✓
Labor induction						✓
Labor augmentation						✓
Mode of delivery						✓
Complications						✓
Medications used						✓

a Replaces mid and late pregnancy surveys if participant enrolls after 32.2 weeks’ gestation.

b Replaces the late pregnancy survey if participant delivers before completing it.

c Anxiety subscale only [55].

d PROMIS: Patient-Reported Outcomes Measurement Information System.

#### EMR Data

The EMR is used to obtain maternal medical history, including vitals (blood pressure and weight) over pregnancy, medication use over pregnancy, PTB interventions (eg, cerclage), and delivery information including date of delivery and GA at delivery (Table 2).

#### Biological Specimens

##### Overview

Biological samples are collected prenatally (up to 3 times during pregnancy; Figure 2 and Table 3), and after delivery. Table 3 lists the biological samples collected, their frequency, and a brief reason for each specimen type. Participants can refuse collection of any biospecimen at any time point and remain in the study.

Each biological sample type is assigned a unique ID number that indicates study ID information as well as visit number, sample type, and aliquot number. No personal identifying information is included in these IDs. Each sample ID is printed as a barcode label that is attached to all specimen collection containers and aliquot containers. All samples and aliquots are scanned into the CLEAR biospecimen database along with their storage location. This database is also used for tracking shipment of specimens for analyses.

**Table 3.** Biological samples collected from pregnant participants in the Center for Leadership in Environmental Awareness and Research study.

Sample type	Time points	Primary purpose
Urine	Up to 3 times during pregnancy (early, mid, and late pregnancy)	Measurement of volatile organic compound metabolites
Blood (serum and plasma)	Up to 3 times during pregnancy (early, mid, and late pregnancy)	Measurement of inflammatory biomarkers
Placenta	After delivery	Epigenetic and transcriptomic analyses

##### Urine

Participants are asked to provide a spot urine sample during a research clinic visit, up to 3 times during pregnancy (early, mid, and late pregnancy visits; Table 3). Urine samples are immediately refrigerated and brought to the HFH Molecular Epidemiology Research Laboratory (MERL). The urine specimen is gently mixed, and urine specific gravity measured with a refractometer before 5 aliquots, each with 1800 µL of urine, are created and stored at −80°C. VOC metabolite measurement has successfully been completed in urine samples in previous studies during pregnancy [56,57], including in our own preliminary work [23]. The VOC metabolites planned to be measured in CLEAR are listed in Table 1.

##### Blood

At research clinic visits, pregnant participants are asked to provide a blood sample, up to 3 times during pregnancy (early, mid, and late pregnancy visits; Table 3). Trained phlebotomists obtain the blood samples (1 ethylene diamine tetraacetic acid and 1 sodium heparin tube). Blood samples are brought back to the HFH MERL for processing. The sodium heparin tube is gently inverted several times and centrifuged at 1300×g at room temperature for 10 minutes. In total, 5 aliquots are created: the first containing 1000 µL of sample and the remaining 4 aliquots each containing 500 µL of sample. Aliquots are stored at −80°C. The ethylene diamine tetraacetic acid tube is processed similarly, except that 1.5 mL of whole blood is aliquoted before centrifuging, and the buffy coat is retained after removal of plasma. Plasma will be used to measure inflammatory biomarkers during pregnancy.

##### Placenta

Placentas are collected after delivery in partnership with the HFH Departments of Pathology and WHS. After delivery of the placenta, WHS staff place the placenta in a placenta collection bucket labeled with the patient’s medical record number, and the bucket is placed in a refrigerator on the labor and delivery floor. The refrigerator has shelves specifically designated for research and pathology. Placentas that require pathological evaluation are retrieved from the HFH Department of Pathology after clinical evaluation. As such, placentas retrieved from pathology are weighed, formalin fixed, and sectioned as part of the clinical evaluation. Placentas not intended for clinical evaluation by the Department of Pathology are picked up daily by the study team and transferred on ice to the MERL for fixation and processing. To the extent possible, processing of placentas that are not sent for clinical pathologic evaluation mirrors that of those sent for pathologic evaluation. This includes weight, length, and thickness measurement, fixation in 10% buffered formalin, and gross evaluation by a histotechnologist. From all fixed placentas, 4 full-thickness sections (1 from each quadrant), 1 section of umbilical cord, and 1 membrane roll are paraffin embedded and stored in cassettes for downstream analyses (eg, DNA and RNA extraction).

### Optional CLEAR Study Activities

As part of the consenting process, participants are asked to opt in or opt out of several optional study activities. Although the participant does have to make a choice to opt in or opt out for each optional study activity, their specific choice does not impact their overall participation in CLEAR. The optional study activities include the following:

- Receipt of text messages from the Twilio system (Twilio Inc), a secure and HIPAA-compliant messaging system that integrates within REDCap
- Collection and storage of leftover clinical samples (eg, urine and blood) from the (1) pregnant participant and (2) child participant
- Collection of newborn screening blood spots (Michigan BioTrust for Health [58])
- Foliage sampling (collaborative work with CLEAR engineering project 1)
- Consent to contact by the CLEAR Community Engagement Core for Healthy Homes (Indoor Home Safety Assessment [59])
- Long-term storage of data and samples for future research

### Primary Health Outcome: PTB

EMR data will be used to determine GA at delivery and define PTB. The “minimal” criteria described by Pennell et al [60] for determining PTB status will be used. GA at delivery will be assigned in accordance with the American College of Obstetricians and Gynecologists guidelines [61]. Our primary analysis will focus on cases defined as PTB if GA at delivery <37 weeks. Secondary analyses will consider the following: (1) continuous GA at delivery, (2) PTB subtypes (eg, extremely preterm if <28 weeks, very preterm if 28 to 32 weeks, and moderate-to-late preterm if 32 to 37 weeks), and (3) whether the PTB was spontaneous (preterm premature rupture of membranes or preterm labor) or indicated for medical reasons.

### Sample Size and Power

The CLEAR birth cohort was designed to examine the association of VOCs with PTB, to examine if VOCs impact risk of PTB via maternal inflammation and placental alterations, and to explore potential sources of VOC exposures. A nested case-control approach was chosen to weigh the high cost of laboratory analyses against the need to recruit patients early enough in pregnancy to accrue repeated and multiple biospecimens; nested case-control designs reduce some costs while having minimal impact on statistical efficiency [62]. Given birth rates at HFH and past birth cohort recruitment [63], we anticipate recruiting 1075 pregnant participants into CLEAR. The PTB rate in Detroit in 2018 was 15.2% [64]. We assume that approximately 15% of the CLEAR cohort will experience PTB; thus, 164 PTB cases are expected. Controls with GA at delivery ≥37 weeks at delivery will be 1:1 frequency matched [65] to cases based on infant sex and maternal race. Frequency matching maintains power [66] yet allows us to still adjust for these variables in the analysis. This sample provides sufficient power for the proposed analyses. For example, based on data from pregnant women in the National Children’s Study [56], SD of VOC metabolites on the log_10_ scale is expected to range from 0.1 to 0.6. With 164 per group for the nested case-control, *α*=.05, 2-tailed tests, and 80% power, an odds ratio of 1.3, 1.6, or 3.5 for a VOC metabolite with an SD of 0.6, 0.3, or 0.1, respectively, could be detected for associations of a VOC metabolite with PTB. For examining the association of VOCs with placental function, with a false discovery rate of 0.05, there is sufficient power (>80%) to detect correlations of ≥0.3 and fold changes of 2.5.

### General Statistical Considerations

A combined directed acyclic graph and change-in-estimate approach will guide selection of confounders and effect modifiers [67-70]. Although a large number of potential confounders will be measured, observational studies are subject to unmeasured confounding: e-values will be used to estimate the effect of unmeasured confounding [71,72]. We will consider methods to account for potential bias or misclassification of PTB case or control status by including the method of assigning GA at delivery (first-trimester ultrasound, second-trimester ultrasound, or date of last menstrual period) in models.

VOC results below the limit of quantification will be imputed as in Wei et al [73]. Multiple testing adjustment for high-dimensional data, such as false discovery rate, will be used [74]. With up to 6 VOCs measured, to reduce the multiple-testing burden, chemical mixture modeling will be used. Potential chemical mixture approaches include Bayesian kernel machine regression, weighted quantile sum regression, and principal components analysis [75]; each has distinct strengths and weaknesses. If more than one method is potentially appropriate, robustness of findings will be examined across different methods and all findings will be reported as in Vuong et al [76].

To examine associations between VOC metabolites and PTB, generalized linear mixed regression models will be used, where the repeated VOC metabolites are the covariates and PTB status (yes or no) is the outcome of interest. Generalized linear mixed model elastic net (glmmlasso), which accounts for mixtures and correlation between repeated measures, will be used to examine associations between VOC metabolites and maternal inflammation [77]. The association of VOC mixtures with DNA methylation will be tested using generalized network-based elastic-net linear mixed model (GELMMnet) with a logit link for β values (equivalent to M values), and associations with gene expression (RNAseq) will be tested using GELMMnet with a Poisson distribution [78]. If significant associations are identified above, we will then test if inflammation and/or placental function mediates associations between VOC metabolites and PTB using a mediation approach for dichotomous outcomes that estimates the natural direct and indirect effects for each individual marker [79], which has been extended to account for repeated measures and high-dimensional mediation effects [80]. To explore neighborhood-level or related factors, clustering or hot spot analysis and geographically weighted regression, a spatial analysis technique that uses nonstationary data (eg, demographic factors and environmental characteristics) to model the local relationship between predictors and an outcome (ie, VOC level), will be used [81,82].

### Engagement and Retention

Multiple strategies have been implemented to promote participant engagement and retention throughout the study. Participants receive personalized communications, including birthday and welcome baby cards, to acknowledge important milestones and maintain an ongoing connection with the study. CLEAR’s partnerships with clinical providers support trust-building and continuity, while study staff are trained to create positive, supportive encounters that help participants feel valued and respected. To help reduce barriers to continued participation, logistical support is offered, including Lyft (Lyft Inc) rides to and from study appointments, parking reimbursement, and a resource flyer tailored to participant needs (eg, mental health and postpartum assistance). Participants also receive prenatal gifts (eg, diaper bag and vanity kit) and age-appropriate gifts for their baby as tokens of appreciation. Collectively, these strategies are designed to foster a supportive study environment, reduce participant burden, and enhance long-term retention.

The consent process incorporates language specific to follow-up, including permission to use publicly available directories, databases (eg, WhitePages), or social media (eg, Facebook; Meta Platforms Inc) to validate contact information if participants are difficult to reach. This ensures that participants are informed of, and comfortable with, strategies to support continued engagement over time. In addition, participants are asked to provide the name and contact information of a family member or close friend that the CLEAR study can reach out to if the participant is lost to follow-up.

## Results

Funding for the study began September 2022. Study recruitment began in November 2023. As of April 22, 2026, a total of 468 pregnant patients have consented to participate in the CLEAR birth cohort. The mean age at consent was 29.9 (SD 6.1) years. On the basis of race information captured in the EMR, most consented participants are Black (346/468, 73.9%), and 16% (75/468) are White, with the remainder reporting either another race, declining to provide race information, or having unknown race. Delivery information is available for 307 (65.6%) participants; of these, 13.7% (42/307) were PTB.

Recruitment is ongoing, with 43.5% (468/1075) of the recruitment goal achieved. Continued efforts to increase recruitment and retention rates will be used, as well as potential expansion of recruitment at other Detroit-based WHS clinics.

## Discussion

### Anticipated Findings

Establishment and study of the CLEAR birth cohort will provide needed data on the role of VOCs in PTB, particularly in older, urban areas such as Detroit, Michigan. Although previous studies have suggested potential associations between VOCs and PTB or other adverse birth outcomes, nearly all have relied on VOC exposures estimated based on residential address, with a single study examining personal exposure to only a single VOC (ie, benzene) [5]. CLEAR will address this limitation via measurement of VOC metabolites in maternal urine across pregnancy. Additionally, better understanding of potential mechanistic pathways, such as impacts on maternal inflammation and on placental function, may reveal areas for interventions to prevent PTB. To date, limited studies have examined these relationships with direct measurements of VOCs and/or in placental tissue; thus, CLEAR will provide unique data on these potential associations. With detailed address information, the CLEAR birth cohort will also allow for the study of potential VOC exposure sources, which are important to identify ways to either decrease exposure or to remediate contaminated areas. Previous studies have identified sources of indoor VOC levels in the homes of children with asthma in Detroit [83]; CLEAR will expand on these previous findings by better understanding sources of direct VOC measurements in pregnant women.

The CLEAR birth cohort addresses limitations of prior studies, importantly by directly measuring VOC exposure via urinary metabolites across pregnancy. Potential limitations, inherent to observational studies, remain. Although CLEAR captures numerous measures of potential confounders, residual confounding remains a potential limitation. This is somewhat mitigated by the proposed use of e-values. Similarly, recruitment bias is a potential issue impacting clinical research studies; because we are recruiting through a health system, we can describe those who do and do not agree to participate in the CLEAR birth cohort (in terms of basic demographic characteristics) which will enable us to address potential bias. Although we propose to measure urinary metabolites and circulating inflammatory biomarkers across pregnancy, not all women enter prenatal care in the first trimester, nor do they complete all study visits, thus missing data are anticipated. Similarly, women who do not deliver at a study hospital or who wish to retain their placenta for personal reasons may not have a placenta for functional measurements. Imputation and/or methods that account for loss-to-follow-up such as inverse probability weighting will be used when feasible. As a postindustrial rust belt city, results will be most generalizable to other similar urban cities, most of which have similarly high PTB rates as Detroit [1,2].

### Future Directions

Establishment of the CLEAR birth cohort, currently focused on PTB, provides opportunities for additional future maternal and child research studies. Although outside the scope of the primary aims of this study, additional maternal and infant data collections are planned, including collection of maternal hair, toenails, and stool for assessment of stress biomarkers, additional environmental exposures, and microbiota, respectively, and neonatal blood spots, urine, and stool for measures of inflammation, metabolomics, and microbiota, respectively. Additional surveys over infancy are also planned to measure other exposures, child health and neurodevelopment, and other lifestyle factors.

## Supplementary material

10.2196/87272 Checklist 1 STROBE checklist.

## Abbreviations

BTEX: benzene, toluene, ethylbenzene, and xylene
CLEAR: Center for Leadership in Environmental Awareness and Research
EDEN: *Etude des Déterminants pré et post natals précoces du développement psychomoteur et de la santé de l'ENfant*
EMR: electronic medical record
GA: gestational age
HFH: Henry Ford Health
HIPAA: Health Insurance Portability and Accountability Act
MERL: Molecular Epidemiology Research Laboratory
PTB: preterm birth
REDCap: Research Electronic Data Capture
VOC: volatile organic compound
WHS: Women’s Health Services
